# Meet Our Editorial Board–*Engineering in Life Sciences*. An Interview With Sascha Beutel Leibniz University Hannover, Hannover, Germany

**DOI:** 10.1002/elsc.70019

**Published:** 2025-03-20

**Authors:** Paul Trevorrow, Sascha Beutel

**Affiliations:** ^1^ Wiley, New Era House Bognor Regis UK; ^2^ Leibniz University Hannover Hannover Germany

## 1 Would You Briefly Explain What Your Research Group is Studying?

I am a diploma chemist by profession, working at the Institute of Technical Chemistry. Despite the name, our work primarily focuses on biotechnology, specifically in bioprocessing. This includes both upstream processing of recommended organisms and downstream processing of products like proteins.

We often produce recombinant enzymes, such as those used for the production of flavors or fragrances like terpenes and flavonoids. This involves recombinantly expressing the necessary proteins or enzymes, isolating them, and applying them in various enzyme technical processes. Our area of expertise encompasses both upstream and downstream processes for prokaryotes, as well as sensor development, including optical sensors, fluorescent sensors, and scattered light sensors.

Our institute has a long‐term collaboration with industry partners. For example, we have developed the SFR vario together with the company PreSens Precision Sensing GmbH, a tablar for online measurements in shake flasks. Additionally, I have been involved in laboratory digitalization projects aimed at creating more intelligent laboratory infrastructures. These efforts have garnered significant attention, particularly through our involvement in the Labvolution, a major biotechnology trade fair in Hanover, previously known as Biotechnica.

Following the retirement of my former supervisor, Thomas Scheper, I have assumed responsibility for additional projects, including mammalian cell culture for monoclonal antibody production and a collaborative project with the United Kingdom focused on the differentiation and large‐scale production of T cells. These projects, while not typically within my usual scope, required continuation and successful completion. Consequently, I have taken on these responsibilities to ensure their advancement.

## 2 How Did You Choose a Career in Biotechnology?

It is important to be at the right place at the right time, particularly in public services or academia. Personally, I had the opportunity to make this decision while I was a PhD student and already a father of two children. My supervisor at the time, Thomas Sheper, offered me a postdoctoral position upon the completion of my thesis. Considering my family responsibilities, I decided that remaining in public service would be beneficial.

Initially, we agreed that I would take on a steady position without the intention to habilitate. This arrangement lasted for approximately 10–12 years. Eventually, my supervisor prompted me to consider habilitation, which I pursued while maintaining my secure position. This unusual but advantageous situation allowed me to build my research group effectively and complete my habilitation without facing stringent time constraints or deadlines.

The primary reason for choosing public service was to balance professional commitments with family life, making it easier to witness my children's growth compared to working in the private sector.

## 3 What are Your Favorite Pastimes Outside of Research?

I enjoy reading and watching movies. I also stay very connected with my grown‐up children, aged 24 and 26, and we do many activities together. Additionally, I like going out with friends.

## 4 What is the First Thing You Do When You Wake up?

When I wake up, the first thing I do is use the restroom. I then prepare a large flask of coffee. As you can see, I always have my coffee nearby, as I require it to function properly in the morning. Following this, I proceed with my usual preparations for the day ahead.

## Data Availability

Data sharing is not applicable to this article as no new data were created or analyzed in this study.

